# A Rare Case of Recurrent Lobular Capillary Hemangioma in an Adult: Diagnosis and Management

**DOI:** 10.7759/cureus.82611

**Published:** 2025-04-20

**Authors:** Nitin Gorwade, Chandulal D Dhalkari, Sachin B Mangalekar, Jeeth J Rai, Shruti H Dhimte

**Affiliations:** 1 Department of Periodontology, Bharati Vidyapeeth (Deemed to be University) Dental College and Hospital, Sangli, IND; 2 Department of Periodontology, Government Dental College and Hospital, Aurangabad, IND

**Keywords:** excision, haemangioma, lobular capillary haemangioma, recurrent, recurrent lobular capillary haemangioma

## Abstract

Hyperplastic lesions of the oral cavity appear pink or reddish-blue in colour and soft to firm in consistency. Its onset is always initiated by a traumatic incident or low-grade chronic irritation. Lobular capillary haemangioma (LCH) is one type of hyperplastic lesion that is most commonly initiated by a traumatic incident.

A 27-year-old male patient presented with a recurrent swelling in the front portion of the lower jaw for one year. The lesion was excised from its identified base. Spontaneous, profuse, pulsatile bleeding was observed post excision. The area was isolated with high vacuum suction to improve visibility. A mid-crestal incision was made at the site of bleeding to access deeper tissues, and a flap was reflected to identify the source of bleeding. Once the source of bleeding was identified, the lesion was cauterised deep within the bone to control bleeding. After the bleeding was controlled, the operated site was sutured with silk sutures. A section of the excised lesion was sent for histopathological examination to determine the nature of the lesion. The procedure aimed to both address the immediate concern of bleeding and provide a definitive diagnosis through histopathological examination for appropriate management.

The histopathological report indicating a rich lobular capillary structure with collagen fibres within the central and peripheral parts of the lesion suggested a diagnosis consistent with intraoral LCH. Intraoral LCH is a rare clinical lesion that may lead to severe bleeding during excision. It's crucial for clinicians to be adequately aware of the lesion and its histopathological details. Considering the high vascular appearance of the lesion, it's advisable to consider a prior incisional biopsy to avoid intraoperative complications. Thus, a biopsy can provide valuable information about the nature of the lesion and guide the surgical approach. When excising the lesion, it's important to remove it from its base to minimise the risk of recurrence. Complete excision from the base, which is located deep within the bone, helps ensure that all abnormal tissue is removed, reducing the chances of recurrence. Overall, a thorough understanding of intraoral LCH, including its histopathological features and potential complications, is essential for clinicians to effectively manage and treat the recurrent lesions.

## Introduction

A lobular capillary haemangioma (LCH) is a rare benign neoplastic lesion seen in the oral cavity. Haemangiomas are a heterogeneous group of clinically benign vascular lesions having similar histologic features [1]. An LCH of the oral mucosa represents inflamed fibrovascular tissues and has been given different names like fibrous inflammatory hyperplasia, giant cell granuloma, palatal papillary hyperplasia, pregnancy epulis, and most commonly, pyogenic granuloma (PG) [2-4]. Histologically, it was described by Angelopoulos as "haemangiomatous granuloma" because of the presence of numerous blood vessels and the inflammatory nature of the lesion [2, 5]. Gingival overgrowth lesions have a broad base of etiologic factors. i.e., inflammatory, drug-induced, idiopathic gingival overgrowth, enlargements associated with systemic disease, neoplastic, and false enlargement.

## Case presentation

**Figure 1:**
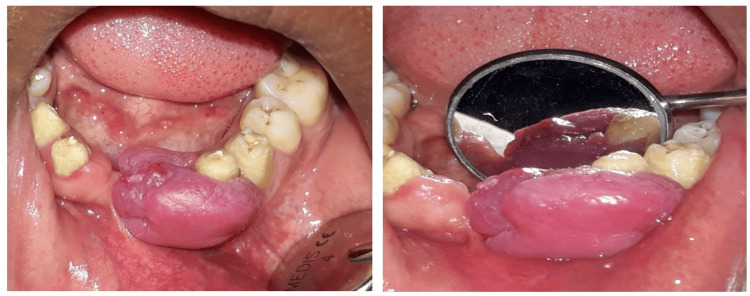
The preoperative image shows a reddish-pink, lobulated, exophytic, pedunculated lesion.

A 27-year-old male patient presented with a chief complaint of swelling in the front portion of the lower jaw (gums) for one year (Figure 1).

The patient presented with a recurrent swelling in the front portion of the lower jaw. The patient gave a history of overgrowth, which first appeared three years back, starting with a very small reddish swelling concerning tooth number 33 because of toothbrush trauma, with the incidence of bleeding, which gradually went on increasing and obliterated the patient’s occlusion; medical history was not significant. The patient reported to a private clinic where they suggested extraction of the involved teeth, i.e., 31, 32, and 41, followed by excision of the lesion. The patient underwent the treatment as per advice; however, the lesion reappeared after nine months. The lesion was re-excised, followed by replacement of missing teeth with a bridge extending from tooth number 42 to tooth number 34 in another private clinic. Histopathological examination was not carried out by the private clinician. However, again after 1.5 years, the patient started noticing the appearance of a small lobulated reddish swelling in the mesial region of tooth number 33, which gradually went on increasing within seven months, causing obliteration in the occlusion and reaching the present size, which was approximately 3x3x2.5 cms in size. The adjustment teeth were non-vital and non-tender on percussion. Overgrowth was lobulated, reddish-pink in appearance, having a pedunculated base and soft to firm in consistency.

Procedure

**Figure 2:**
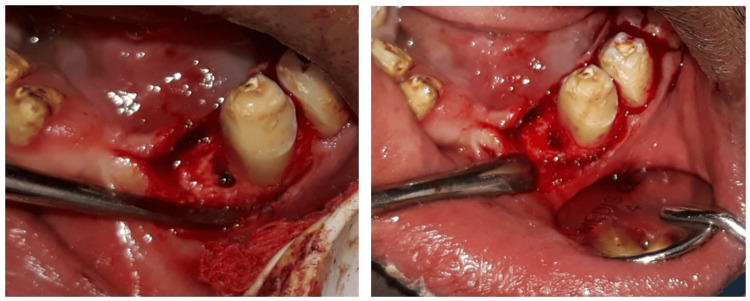
Intraoperative image showing flap reflection and the source of bleeding

Local anaesthesia was administered, and the stalk of the lesion was tied with a silk suture to identify its base. The lesion was excised using a 15D blade at its identified base. Spontaneous, profuse, pulsatile bleeding was observed post excision. The area was isolated with high vacuum suction to improve visibility. A mid-crestal incision was made at the site of bleeding to access deeper tissues, and a flap was reflected to identify the source of bleeding (Figure 2).

**Figure 3:**
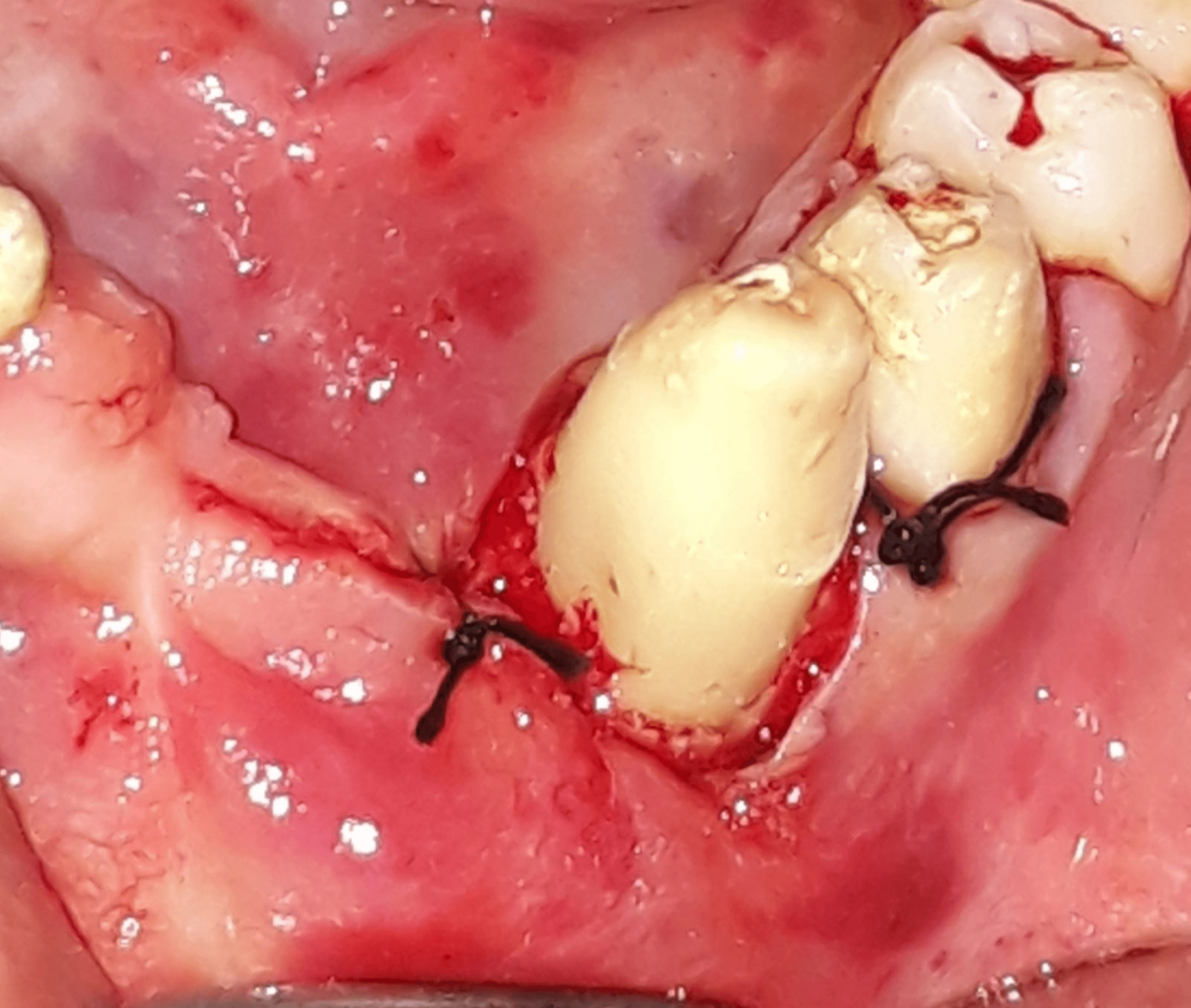
A 3-0 silk suture was used to approximate the flap.

Once the source of bleeding was identified, the lesion was cauterised deep within the bone to control bleeding. After the bleeding was controlled, the operated site was sutured with silk sutures (Figure 3).

**Figure 4:**
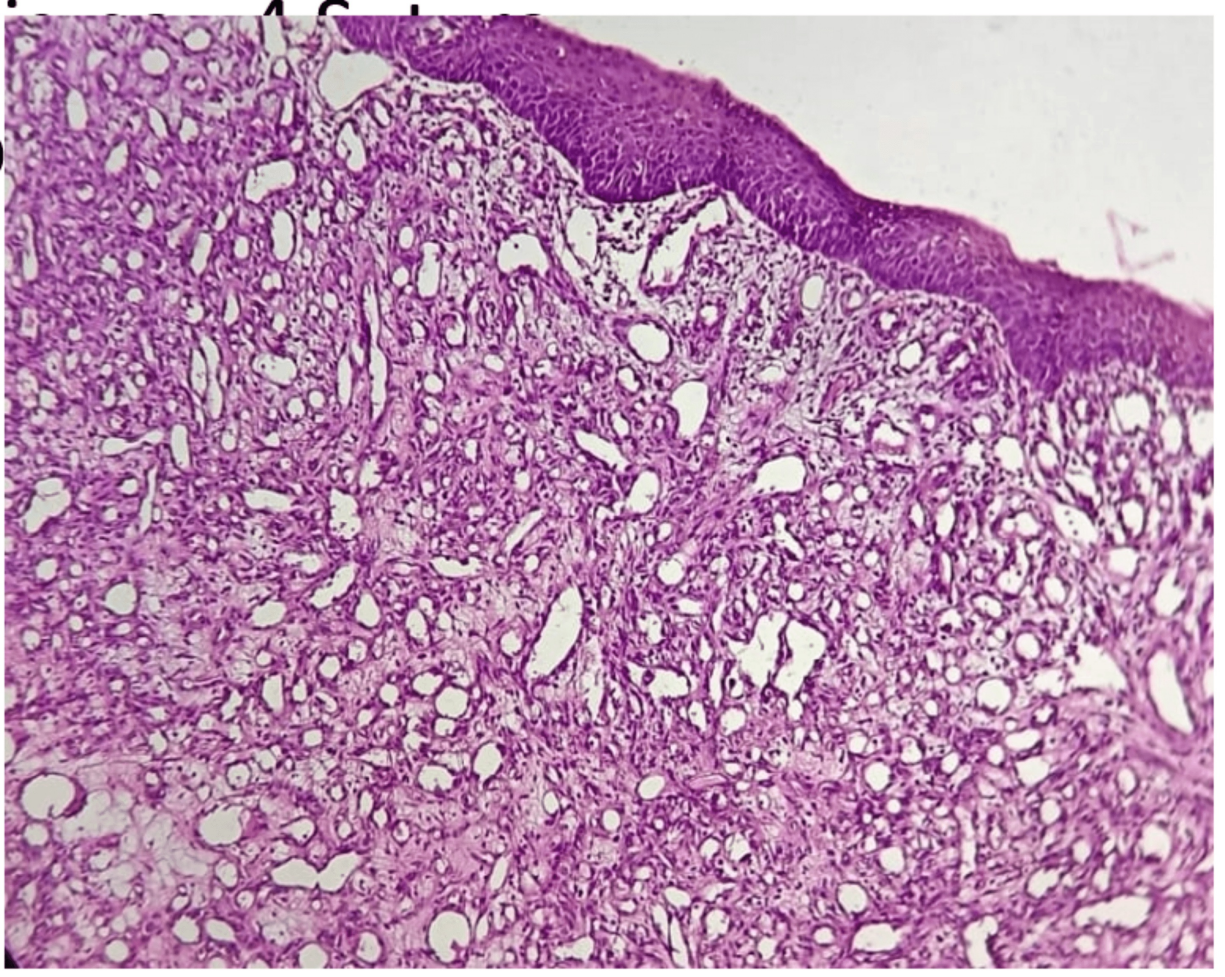
Histopathological image with H&E staning under 10X magnification

**Figure 5:**
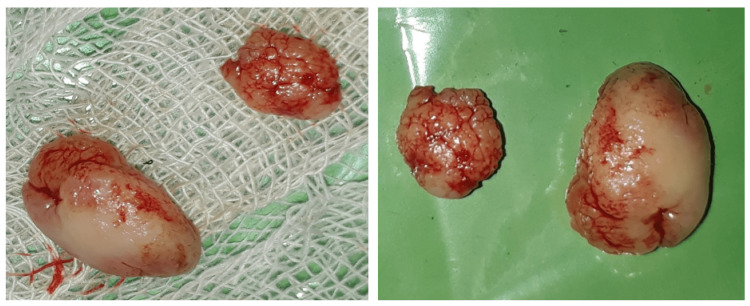
Excised lesion showing two pieces with a lobular appearance

A section of the excised lesion was sent for histopathological examination; the histological image showed aggregates of lobulated blood vessels in the connective tissue stroma (Figures 4, 5), and the patient was called back after seven days.

Outcome

**Figure 6:**
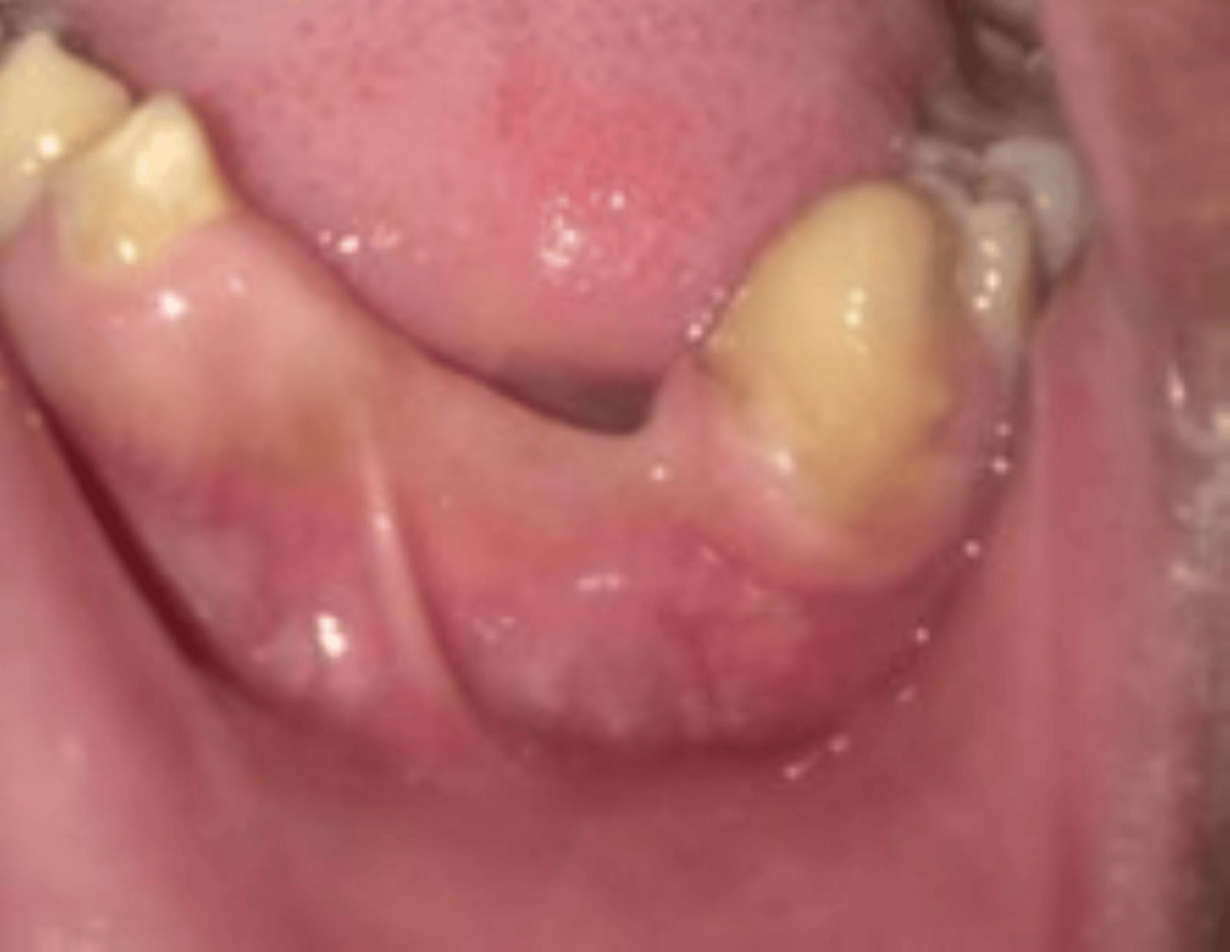
Postoperative image obtained after three years showing well adapted gingival tissue

Postoperative healing observed on the recall visit was uneventful. Currently, the patient is fine with no signs of recurrence after three years of follow-up (Figure 6).

## Discussion

Low-intensity chronic irritation may lead to the development of reactive hyperplastic lesions in the oral cavity [2, 6]. Haemangiomas are a type of hyperplastic lesion which are characterised by three phases, i.e., endothelial cell proliferation, rapid growth, and spontaneous involution. The most common causative factor for extragingival pyogenic granuloma (PG) is trauma. About 30% of the lesions will report with trauma, chronic irritation, defective fillings, food impaction, and toothbrush trauma [2, 7, 8-13]. The present case had a history of toothbrush trauma before the onset of the lesion. Clinical appearance showed a lesion that was lobulated, exophytic, having a pedunculated or sessile base. PGs generally are soft, painless, and deep red to reddish purple in colour and haemorrhagic on slight irritation [2, 3]. Young PGs are highly vascular in appearance because they are composed predominantly of hyperplastic granulation tissue in which capillaries are prominent. Thus, minor trauma to the lesion may cause considerable bleeding, whereas older lesions tend to become more collagenised and pink [2, 7, 14]. It was reported by Epivatianos et al. that the two types of PG were clinically different, showing that foci of fibrous maturation were present in 15% of non‑LCH PG but were totally absent in LCH PG [2, 15]. They found that the LCH PG occurred more frequently (66%) as a sessile lesion, whereas the non‑LCH PG mostly occurred as a pedunculated lesion (77%) [2, 15]. Usually, a PG will be given as a provisional diagnosis until the histopathological report confirms the final diagnosis. The lobular area of the LCH-type PG contains a higher number of blood vessels with small luminal diameters than does the central area of non‑LCH PG. It is possible that these differences may suggest that these two histological types of PG may represent different biological behaviours [2, 15]. The presence of blood vessels with different luminal diameters in the lobular area of LCH PG and in the central area of non‑LCH PG may be because of different pathogenic factors influencing their development [2, 7, 15]. A higher female-to-male ratio was seen in the study reported by Olawoyin et al. [16]. The size varies in diameter from a few millimetres to several centimetres. PG reaches its full size within weeks or months and rarely exceeds 2.5 cm in diameter [2, 17]. The current case size noted was more than 2.5 cm, and two recurrences were noted. Differential diagnosis of inflammatory fibrous hyperplasia, giant cell fibroma, peripheral giant cell granuloma, and peripheral ossifying fibroma may be considered, as all the below-mentioned lesions are reactive hyperplastic in nature (Table 1) [18, 19].

**Table 1:** Differential diagnoses to be considered Sources: [18,19]

Lesion	Clinical features	Histologic features
Peripheral fibroma	A firm, pink, uninflamed mass; it grows from below the free gingival margin or interdental papilla and is painless in nature. As age advances, it may show calcifying, cementifying and ossifying lesions.	Prominent fibromyxoid stroma, variable cellularity, a whorled or storiform pattern of arrangement of the cellular elements, lack of significant inflammation or vascularity, and complete absence of calcification and/or odontogenic islands. Fibroma may show additional foci of calcification (peripheral calcifying fibroma), foci of cementicles (peripheral cementifying fibroma), or trabeculae of bone (peripheral ossifying fibroma).
Angiogranuloma/pyogenic granuloma	A painless, sessile/pedunculated tissue, with a size ranging from a few mm to cm, has a colour appearance of deep red or bluish red, as they are highly vascular and composed of hyperplastic granulation tissue with profuse blood capillaries.	Histologically, the stratified squamous epithelium is thickened, with prominent rete pegs and some degree of intracellular and extracellular oedema, prominent intercellular bridges and leukocytic infiltration.
Peripheral giant cell granuloma	They occur particularly in the anterior region in young patients or in the posterior mouth during the mixed dentition phase and in adults. They are very aggressive lesions with significant growth potential. The high vascularity of these lesions can be understood by their purplish-red colour and tendency to bleed. They also tend to penetrate interdentally, and erosion of adjacent bone along with separation of adjacent teeth is a common occurrence.	A microscopic view shows a nodular arrangement of giant cell tissue separated by fibrous septa. The giant cell tissue consists of a mixture of multinucleated and mononuclear giant cells against extravasated red blood cells. Some sinusoidal spaces and capillary vessels are usually present. The fibrous stroma may be loose or dense and contains large, thin-walled vascular structures. Heavy deposits of hemosiderin are also seen within the giant cell tissue and the surrounding fibrous component.
Giant cell fibroma	A solitary, greyish-brown sessile overgrowth, firm and fibrotic with a rough, pebbly surface and corrugated gingival margin.	Shows a fibrous mass covered by hyperparakeratotic stratified squamous epithelium with elongated and pointed rete ridges. There is the presence of mononuclear or binuclear stellate giant fibroblasts dispersed in the subepithelial fibrous connective tissues.

Incidence of intraoral LCHs varies from 0.5 to 1.0% of all intraoral neoplasms [20, 21]. All the reactive lesions have a few similar clinical features but are distinct in their histologic appearance. The most common treatment modality of haemangioma is surgical excision of the lesion, with or without ligation of vessels and embolisation [20, 21]. Surgical management should be done with caution because of the possibility of bleeding intraoperatively and postoperatively. Recently developed treatment modalities include steroid therapy, electrosurgery, neodymium-doped yttrium aluminium garnet (Nd:YAG) laser, carbon dioxide (CO₂) laser, cryosurgery, and sclerotherapy [20, 21]. Considering the two-time recurrence of the lesion and high vascular appearance in view, the current case was planned and executed by keeping a combined surgical approach, i.e., conventional scalpel excision followed by deep cauterisation till the point of the source of bleeding. By employing deep cauterisation to target the bleeding source deep within the bone, which likely ensured more thorough removal of the lesion and its vascular components, thus reducing the chances of recurrence. Cauterisation serves to destroy tissue and seal off blood vessels, which can be crucial in preventing recurrence by addressing the source of bleeding and potentially eliminating residual cells that could contribute to regrowth. Additionally, by reaching deep into the bone, we had successfully reduced the risk of leaving behind any remnants of the lesion that could promote recurrence. This combined approach not only addresses the visible aspect of the lesion through excision but also tackles the underlying vascular elements that may contribute to its persistence. By effectively addressing both aspects of the lesion, we have optimised the chances of preventing further recurrence and promoting successful resolution. The haemangioma has an excellent prognosis as it does not undergo malignant transformation or recur after adequate removal [17]. Considering the two-time recurrence of the lesion, the case was properly planned by keeping a combined surgical management approach as per the description of the case report. Currently, the patient is fine with no signs of recurrence after three years.

## Conclusions

Intraoral LCH is a rare and uncommon intraoral soft tissue lesion which may lead to severe bleeding during excision. Hence, surgical excision should be carried out with great caution. Early diagnosis, followed by confirmation with the help of biopsy, is essential to determine the clinical behaviour of the lesion. Clinicians should be adequately aware of the lesion, and surgery should be carried out by taking the utmost precautions.
